# Baduanjin exercise can alleviate anxiety and depression of patients with COVID-19 in Square cabin hospital: A cross-sectional survey

**DOI:** 10.1097/MD.0000000000026898

**Published:** 2021-08-13

**Authors:** Xiao-Bo Zhang, Jin-Long Zhang, Ming-Xia Li, Ying-Pu Yuan, Juan Sun

**Affiliations:** Department of Neurology, The First People's Hospital of Changde City, Changde, Hunan Province, China.

**Keywords:** COVID-19, baduanjin exercise, square cabin hospital, anxiety, depression

## Abstract

To investigate the anxiety and depression of patients with the coronavirus disease 2019 (COVID-19) who participated in Baduanjin exercise.

From February 20, 2020 to March 7, 2020, the Hospital Anxiety and Depression scale (HAD) were used to investigate the anxiety and depression levels of patients with COVID-19 who participated in Baduanjin exercise. Ninety one questionnaires were received, including 40 males and 51 females. Stepwise regression analysis was used to analyze the effects of related factors on anxiety and depression levels.

In Square cabin hospital, 91% of patients participated in Baduanjin exercise had no obvious anxiety and 82% had no obvious depression. The scores of anxiety and depression of female patients were significantly higher than that of male patients. Bachelor degree or above with low scores for anxiety and depression. The frequency of Baduanjin exercise was negatively correlated with anxiety and depression score.

The development of Baduanjin exercise has a certain positive influence on the COVID-19 patients in the Square cabin hospital, which is conducive to alleviate anxiety and depression symptoms of the patients.

## 1 Introduction

COVID-19 is a new infectious disease, which has the characteristics of human-to-human transmission, long latency and high mortality.^[1]^ Up to now, there is still a lot of uncertainty about the origin, nature and process of the disease. Therefore, people are extremely lack of understanding of it. For the time being, there is no specific cure for the disease, which also aggravates people's panic and fear of COVID-19. Therefore, it is necessary to find suitable ways to alleviate patient's anxiety and depression.

As a traditional qigong exercise, Baduanjin exercise has the advantage of easy learning and no need for physical strength, which can relax the mind and promote sleep.^[2]^ Some studies have also shown that Baduanjin exercise has a significant effect in reducing anxiety and depression.^[3]^ The Square cabin hospital where the patients are located is transformed by large indoor stadiums and stadiums,^[4]^ which is suitable for the treatment of mild COVID-19 patients. In order to alleviate the anxiety and depression of patients, enrich their lives and promote their early recovery, medical staff organized Baduanjin exercise.

Through literature search, few studies have been reported on the effect of Baduanjin exercise on anxiety and depression levels in patients with COVID-19. The study is to investigate the anxiety and depression of patients with COVID-19 who participated in Baduanjin exercise during hospitalization in Square cabin hospital. It is of great significance to the later targeted psychological intervention and promoting the rehabilitation of patients.

## 2 Methods

### 2.1 Respondents

In Wuhan Huangpi Square cabin hospital, under the premise that COVID-19 patients voluntarily participated in Baduanjin exercise, professional medical staff organized Baduanjin exercise four times a day for 20 min each time. Using the convenient sampling method, a questionnaire survey was conducted on 91 COVID-19 inpatients (with complete patient files) who met the enrollment criteria in Square cabin hospital, Huangpi District, Wuhan City, Hubei Province from February 20, 2020 to March 7, 2020. A total of 100 questionnaires were sent out in this survey, 91 valid questionnaires were collected. The study subjects were selected according to the following criteria:

1. patients over 18 years old;
2. patients who were diagnosed as COVID-19 with self-care ability;
3. patients with mild and common clinical types;
4. without cardiac, pulmonary or renal dysfunction;
5. no history of mental illness and other history of malignant tumor.

Exclusion criteria:

1. refusing to participate in this study;
2. patients who do not have the ability to take care of themselves.

Ethical approval was issued by the Ethics Committee of The First People's Hospital of Changde City and Wuhan mobile cabin hospital, and all the respondents had signed an informed consent before the study was initiated.

### 2.2 Research tools

After being approved by the research ethics board, the researchers conducted a face-to-face questionnaire survey with the informed consent of the patients. The research tools are as follows:

1. General information questionnaire: including gender, age, education level, occupation and frequency of participating in Baduanjin exercise.
2. HAD scale: including anxiety and depression items, each with 7 items.^[5]^ Each item is a four-level score (0–3 points). The two sets of projects are superimposed separately to get their respective total scores. The total score of 0–7 represents normal; the total score of 8–10 indicates that there may be anxiety / depression; 11–21 indicates the diagnosis of anxiety and depressive symptoms.^[6]^

### 2.3 Statistical analysis

EXCEL was used to establish a database for real-time dual input. SPSS 23.0 statistical software was used for statistical analysis. The general data and anxiety and depression scores of COVID-19 patients were expressed as median (quartile interval). Mann–Whitney *U* test was used for gender. Kruskal–Wallis *H* test was used for other comparisons. Stepwise regression was used to analyze the effect of related factors on anxiety and depression levels.

## 3 Results

### 3.1 Anxiety and depression scores of patients (Table 1)

The scores of anxiety and depression were (4.47 ± 3.24) and (4.34 ± 3.74), respectively. The scores of anxiety and depression were at the normal level. 8.79% of the patients were suspicious or diagnosed with anxiety, and 17.58% were suspicious or diagnosed with depression.

**Table 1:** Anxiety and depression scores of patients (n = 91).

	Normal	Suspicious	Diagnosis	Incidence rate	Average
Anxiety	83	6	2	8.79%	4.47
Depression	75	6	10	17.58%	4.34

### 3.2 General data of COVID-19 patients and scores of anxiety and depression (Tables 2 and 3)

Among the anxiety scores, gender and education level were statistically significant (*P* < .05 or *P* < .05). The score of female was 5 (4) higher than that of male, and the score of senior high school and college education was 6 (4) higher than that of others. Among the depression scores, gender, education level and frequency of participating in Baduanjin exercise were statistically significant (*P* < .05 or *P* < .05). The higher score was 4 (4) for females, 4 (6.75) for senior high school and college education, and 11 (5.5) for those who did not participate in Baduanjin exercise.

**Table 2:** Analysis of general data and anxiety level of COVID-19 patients.

Project	Grouping	Number of cases	Anxiety M(Q)	z/H	*P*
Gender	Male	40	3 (5)	−3.05	.00
	Female	51	5 (4)		
Age (years)	Under the age of 20	0		2.34	
	20–40	35	4 (6)		.50
	40–60	50	4.5 (5)		
	60–80	5	5 (4)		
	Over 80 years old	1	2 (0)		
Occupation	Personnel of enterprises and institutions	14	3 (3.5)	4.11	.39
	Business and service personnel	10	4 (4.5)		
	Freelance worker	33	5 (5)		
	Agricultural, forestry, animal husbandry and fishery production personnel	5	6 (2.5)		
	Others	29	4 (6)		
Degree of education	Junior high school and below	44	4 (5)	4.84	.085
	High school and college	30	6 (4)		
	University and undergraduate	17	3 (3)		
	Master's degree or above	0			
Frequency of participation in Baduanjin exercise	0	5	6 (4.5)	1.64	.65
	1	16	4.5 (5.75)		
	2	53	4 (5)		
	More than 3 times	17	4 (5)		

**Table 3:** Analysis of general data and depression level of COVID-19 patients.

			Depression score		
Project	Grouping	Number of cases	M (Q25, Q75)	z/H	*P* value
Gender	Male	40	2.5 (5.75)	−1.93	.05
	Female	51	4 (4)		
Age (years)	Under the age of 20	0		2.6	
	20–40	35	3 (5)		.46
	40–60	50	3 (5.25)		
	60–80	5	4 (9.5)		
	Over 80 years old	1	11 (0)		
Occupation	Personnel of enterprises and institutions	14	1.5 (3.5)	5.87	.21
	Business and service personnel	10	3 (6.5)		
	Freelance worker	33	4 (5)		
	Agricultural, forestry, animal husbandry and fishery production personnel	5	4 (3.5)		
	Other	29	4 (8)		
Degree of education	Junior high school and below	44	3.5 (5.75)	6.73	.04
	High school and college	30	4 (6.75)		
	University and undergraduate	17	2 (2.5)		
	Master's degree or above	0			
Frequency of participation in Baduanjin exercise	0	5	11 (5.5)	13.15	.00
	1	16	5 (4.5)		
	2	53	3 (5)		
	More than 3 times	17	3 (3.5)		

### 3.3 Correlation analysis between different factors and anxiety and depression scores (Tables 4 and 5 and Figure 1)

The scores of anxiety and depression were taken as dependent variables, and the general data as independent variables, that is, sex, age, education level, frequency of participating in Baduanjin exercise and anxiety and depression were analyzed respectively. The results of stepwise regression analysis (*P* < .05 or p approximately < .05) showed that gender was related to anxiety, and the score of females was higher than that of males. In the depression score, gender, education level and frequency of participating in Baduanjin exercise were related to the score of depression. Among them, the score of males was lower than that of females, and the score of college or undergraduate degree was lower than that of other educations. The frequency of participating in Baduanjin exercise was negatively correlated with the score of depression.

**Table 4:** Results of stepwise regression analysis of influencing factors of anxiety score.

Independent variable	Partial regression coefficient	Standard error	T value	*P* value
Constant	3.41	0.57	5.98	<.00
Gender				
Female	2.00	0.65	3.1	.00
Degree of education				
High school and college	0.59	0.72	0.82	.42
University and undergraduate	−1.35	0.87	−1.55	.12
R^2^ = 0.15	Adjusted R^2^ = 0.12	*P* = .49		

**Table 5:** Results of stepwise regression analysis of influencing factors of depression score.

Independent variable	Partial regression coefficient	Standard error	T value	*P* value
Constant	9.32	1.56	5.98	<.00
Gender				
Female	1.43	0.73	1.96	.05
Degree of education				
High school and college	0.57	0.82	0.70	.49
University and undergraduate	−1.73	0.95	−1.82	.07
Frequency of participating in Baduanjin exercise				
1	−4.38	1.73	−2.54	.01
2	−6.31	1.55	−4.06	.00
3	−6.38	1.69	−3.77	.00
R^2^ = 0.27	Adjusted R^2^ = 0.22	*P* = .00		

**Figure 1:**
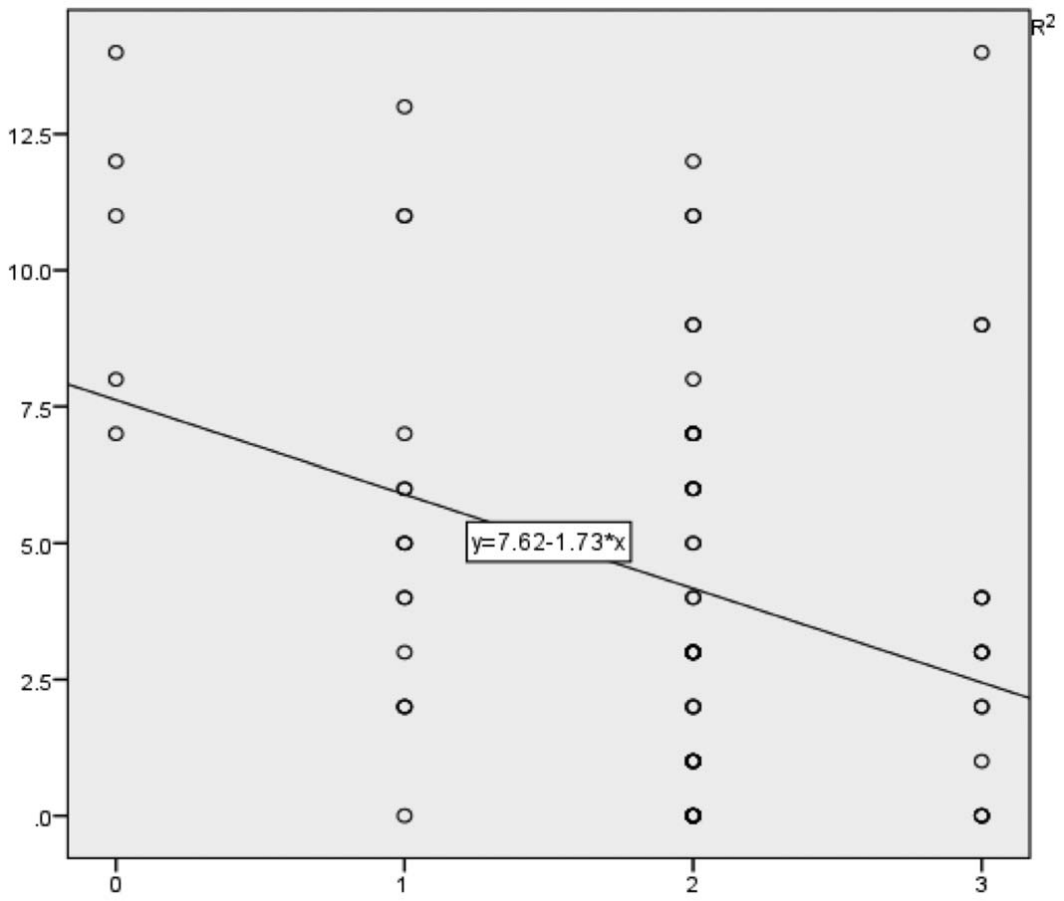
Linear analysis trend chart of the correlation between the frequency of participating in Baduanjin exercise and the scores of depression.

## 4 Discussion

According to the epidemiological survey,^[7]^ the overall curve of COVID-19 showed an epidemic pattern, and the mild patients treated in the Square cabin hospital were restricted in their normal life, could not meet with their relatives and friends, and their social and psychological support were not satisfied. This study found that the anxiety and depression level of mild COVID-19 patients was generally normal during hospitalization in the Square cabin hospital, which may be related to the nature of the Square cabin hospital. Some studies have shown that family income can affect the psychological state of patients.^[8]^ The financial burden of patients with low family income is high, which is one of the main reasons affecting the treatment of inpatients. During the COVID-19 epidemic, the Square cabin hospital had public welfare, and all patients could participate in the treatment free of charge, without considering related issues such as economic costs. In addition, the Square cabin hospital was equal, and all people had the same amount of food and clothing, which was distributed according to needs, eliminating the gap in the identity and wealth of the crowd, so most patients had no obvious anxiety and depression. However, a small number of patients have anxiety and depression, which may be related to COVID-19 's unknown and patients worried about the prognosis of the disease.

Our results show that female, high school and junior college students who do not participate in Baduanjin exercise have higher scores on anxiety and depression. Gender is an important factor affecting the anxiety level of COVID-19 patients, gender, education level and frequency of Baduanjin exercise are important factors affecting the depression level of COVID-19 patients, which are statistically significant in multiple stepwise regression analysis and rank sum test analysis. Age and occupation were not statistically significant in the two analyses, and the detailed analysis was as follows:

### 4.1 Effect of Baduanjin exercise on anxiety and depression

Referring to the literature, there are few domestic studies on the relationship between Baduanjin exercise and anxiety and depression symptoms. Foreign studies^[9]^ have shown that exercise is related to the levels of anxiety and depression, and exercise has been proved to be an economical and effective adjuvant therapy. An appropriate amount of physical activity plays an irreplaceable role in improving physical quality, life satisfaction, cognitive function and mental health. Most of the patients hospitalized in Square cabin hospital are mild or ordinary, and they are generally treated with oral drugs and have the ability to move independently. In order to alleviate patients’ tension about unfamiliar environment and enrich their spare time life, medical workers led the patients to do Baduanjin exercise. Exercise can reduce anxiety and depression by increasing the level of monoamine in the human body.^[10]^ The frequency of participating in Baduanjin exercise is negatively correlated with the level of anxiety and depression of patients. Patients with high participation have low scores of anxiety and depression. We think that physical exercise through Baduanjin exercise can relieve the mood of patients.

### 4.2 Effect of gender on the scores of anxiety and depression

Gender was found to be an important factor affecting anxiety and depression scores by stepwise regression analysis. Women scored significantly higher than men, meaning that women were more likely to experience anxiety and depressive symptoms, which is consistent with Pranas’ study.^[11]^ The main reason for this may be related to the changes in female hormone levels.^[12]^ In female, reproductive hormones have greater mobility throughout the life cycle, increased sensitivity to catecholamines, and enhanced the consolidation of emotional memory. Moreover, females are more likely to meditate than men, which is an important risk factor for anxiety and depression.^[13]^ In addition, female may have more delicate emotions than male and are more likely to be melancholic and worried about the future. Moreover, we believe that during hospitalization at Square cabin hospital, female patients may be more likely to miss their families and worry about the progression of the disease, making them more anxious and depressed than male.

### 4.3 Effect of education level on the scores of anxiety and depression

Education level is an important factor affecting the scores of anxiety and depression. The scores of anxiety and depression from low to high were: university and undergraduate, junior high school and below, high school and college. Some studies have shown that a relatively poor educational background is an important factor in anxiety, mainly because people with higher education may increase their sense of self-efficacy and personal control, and at the same time, people with higher education tend to have higher socio-economic status. therefore, they may be rich in resources and have more correct understanding of the disease. The survey of this study shows that the score of people with university and undergraduate is the lowest, which is consistent with foreign research,^[14]^ but the difference is that people with high school and college are higher than those with junior high school and below, which may be related to two reasons: first, the sample size is small. second, because people with junior high school and below degrees are affected by their educational background, they lack knowledge of COVID-19 and know less about new media and other online platforms. On the other hand, people with high school and college have limited knowledge reserves and have many channels to get information in time, but the network information lacks the correct screening and discrimination ability. this can explain why people with high school and college have higher scores of anxiety and depression than those with junior high school and below degrees.

Of course, we also need to acknowledge that this study also has some limitations:

1. the sample size is relatively small and the survey is limited to one hospital.
2. the individual differences and psychological conditions of the sample
3. the influence of environmental and cultural factors on the individual.

## 5 Conclusion

The development of Baduanjin exercise has a certain positive impact on patients with COVID-19 in Square cabin hospital, which is conducive to reducing anxiety and depression symptoms and relieving psychological pressure.

## Acknowledgments

The authors wish to thank all respondents for their contributions to the study.

## Author contributions

**Conceptualization:** Juan Sun, Jin-Long Zhang.

**Data curation:** Juan Sun, Ming-Xia Li.

**Formal analysis:** Xiao-Bo Zhang.

**Funding acquisition:** Xiao-Bo Zhang.

**Investigation:** Juan Sun, Ming-Xia Li.

**Project administration:** Juan Sun, Xiao-Bo Zhang.

**Supervision:** Xiao-Bo Zhang.

**Writing – original draft:** Xiao-Bo Zhang, Ying-Pu Yuan.

**Writing – review & editing:** Juan Sun, Xiao-Bo Zhang, Ying-Pu Yuan.

## References

[1] Sun P Lu X Xu C Sun W Pan B. Understanding of COVID-19 based on current evidence [published online ahead of print, 2020 Feb 25]. J Med Virol 2020;10.1002/jmv.25722. 10.1002/jmv.25722 PMC7228250 32096567

[2] Chen MC Liu HE Huang HY Chiou AF. The effect of a simple traditional exercise programme (Baduanjin exercise) on sleep quality of older adults: a randomized controlled trial. Int J Nurs Stud 2012;49:265–73. 21963235 10.1016/j.ijnurstu.2011.09.009

[3] Zou L Yeung A Quan X. Mindfulness-based Baduanjin exercise for depression and anxiety in people with physical or mental illnesses: a systematic review and meta-analysis. Int J Environ Res Public Health 2018;15:321. 10.3390/ijerph15020321 PMC5858390 29439556

[4] Novel Coronavirus Pneumonia Emergency Response Key Places Protection and Disinfection Technology Team. Chinese center for disease control and prevention. Zhonghua Yu Fang Yi Xue Za Zhi 2020;54:E006.

[5] Zigmond AS Snaith RP. The hospital anxiety and depression scale. Acta Psychiatr Scand 1983;67:361–70. 6880820 10.1111/j.1600-0447.1983.tb09716.x

[6] Djukanovic I Carlsson J Arestedt K. Is the Hospital Anxiety and Depression Scale (HADS) a valid measure in a general population 65–80 years old? A psychometric evaluation study. Health Qual Life Outcomes 2017;15:193. 28978356 10.1186/s12955-017-0759-9 PMC5628437

[7] Ahn DG Shin HJ Kim MH. Current status of epidemiology, diagnosis, therapeutics, and vaccines for novel coronavirus disease 2019 (COVID-19). J Microbiol Biotechnol 2020;30:313–24. 32238757 10.4014/jmb.2003.03011 PMC9728410

[8] Kim D. Relationships between caregiving stress, depression, and self-esteem in family caregivers of adults with a disability. Occup Ther Int 2017;2017:1686143 Published 2017 Oct 17. 29114184 10.1155/2017/1686143 PMC5664279

[9] Carek PJ Laibstain SE Carek SM. Exercise for the treatment of depression and anxiety. IntJ Psych Med 2011;41:15. 10.2190/PM.41.1.c 21495519

[10] Wegner M Helmich I Machado S Nardi AE Arias-Carrion O Budde H. Effects of exercise on anxiety and depression disorders: review of meta- analyses and neurobiological mechanisms. CNS Neurol Disord Drug Targets 2014;13:1002–14. 24923346 10.2174/1871527313666140612102841

[11] Pranas Serpytis Petras. Gender-based differences in anxiety and depression following acute myocardial infarction. Arquivos Brasileiros De Cardiologia 2018. 10.5935/abc.20180161 PMC6248233 30156607

[12] Altemus M Sarvaiya N Neill Epperson C. Sex differences in anxiety and depression clinical perspectives. Front Neuroendocrinol 2014;35:320–30. 24887405 10.1016/j.yfrne.2014.05.004 PMC4890708

[13] Altemus M. Sex differences in depression and anxiety disorders: potential biological determinants. Horm Behav 2006;50:534–8. 16920114 10.1016/j.yhbeh.2006.06.031

[14] Pham T Jetté N Bulloch AGM Burton JM Wiebe S Patten SB. The prevalence of anxiety and associated factors in persons with multiple sclerosis. Mult Scler Relat Disord 2018;19:35–9. 29125968 10.1016/j.msard.2017.11.003

